# P-2177. Characteristics and Healthcare Resource Utilization of Adults Hospitalized with hMPV and RSV in Southern California between 2011 and 2024

**DOI:** 10.1093/ofid/ofaf695.2340

**Published:** 2026-01-11

**Authors:** Emily Rayens, Lina S Sy, Lei Qian, Bradley Ackerson, Jaejin An, Yi Luo, Xuan Huang, Jennifer H Ku, Punam P Modha, Radha M Bathala, Sudhir Venkatesan, Lisa Glasser, Daniel Molnar, Richard McNulty, Ajoke Sobanjo-ter meulen, Chengbin Wang, Hung Fu Tseng

**Affiliations:** Kaiser Permanente Southern California, Pasadena, CA; Kaiser Permanente Southern California, Pasadena, CA; Kaiser Permanente Southern California, Pasadena, CA; Kaiser Permanente Southern California, Pasadena, CA; Kaiser Permanente Southern California, Pasadena, CA; Kaiser Permanente Southern California, Pasadena, CA; Kaiser Permanente Southern California, Pasadena, CA; Kaiser Permanente Hawaii, Honolulu, HI; Kaiser Permanente of Southern California, Pasadena, California; Kaiser Permanente Southern California, Pasadena, CA; Medical and Payer Evidence Statistics, BioPharmaceutical Medical, AstraZeneca, Cambridge, UK, Cambridge, England, United Kingdom; AstraZeneca, Wilmington, DE; AstraZeneca, Barcelona, Catalonia, Spain; Medical Affairs, Vaccines and Immune Therapies Unit, AstraZeneca, Cambridge, UK, Cambridge, England, United Kingdom; Astra Zeneca, Seattle, Washington; AstraZeneca, Barcelona, Catalonia, Spain; Kaiser Permanente Southern California, Pasadena, CA

## Abstract

**Background:**

Human metapneumovirus (hMPV) and respiratory syncytial virus (RSV) are among the leading causes of acute respiratory infection. While RSV disease burden is well described, the overall burden and clinical significance of hMPV are yet to be fully explored. We evaluated the risk of severe outcomes in adults hospitalized with hMPV compared to RSV at Kaiser Permanente Southern California.

**Figure:**
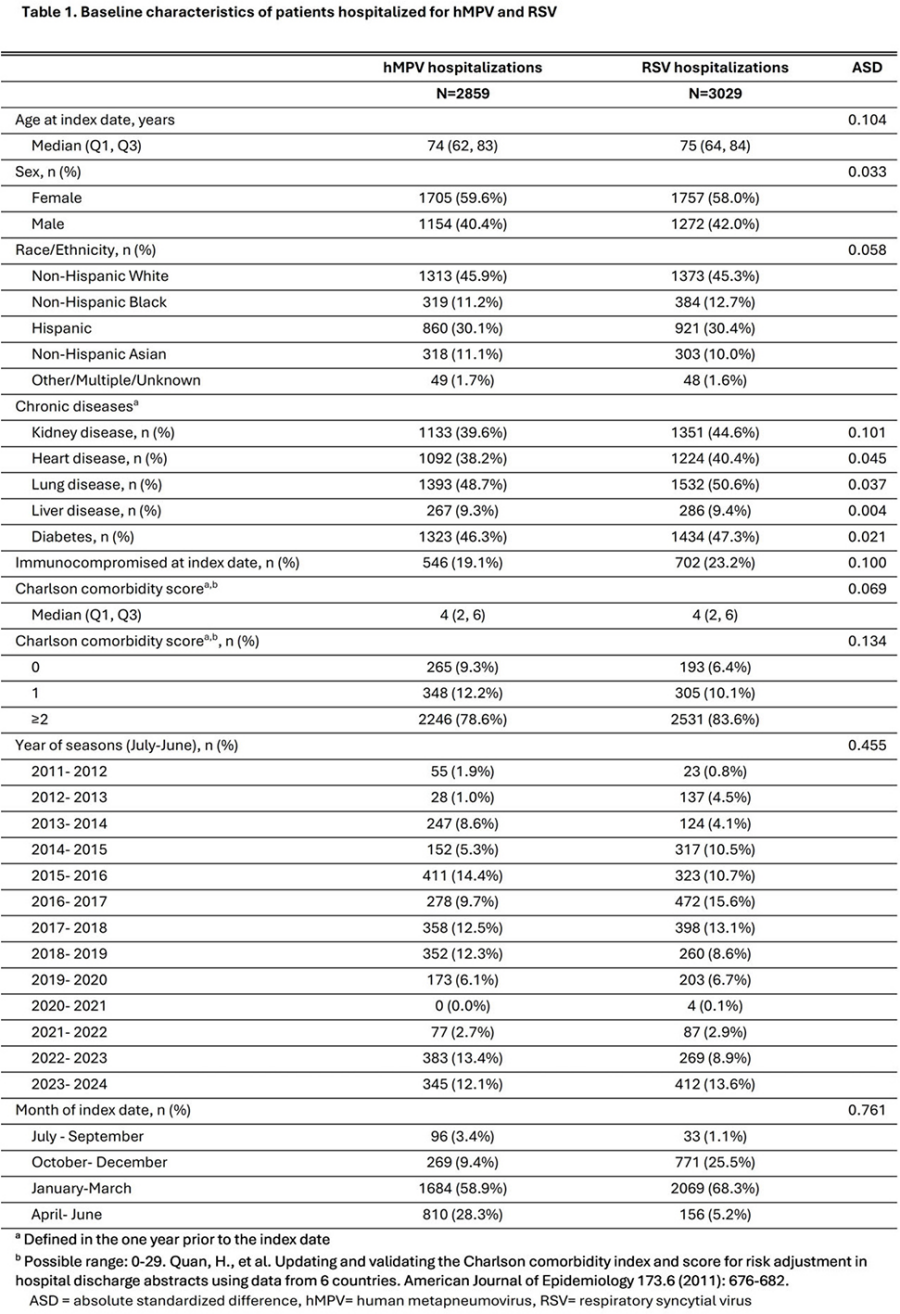

**Figure:**
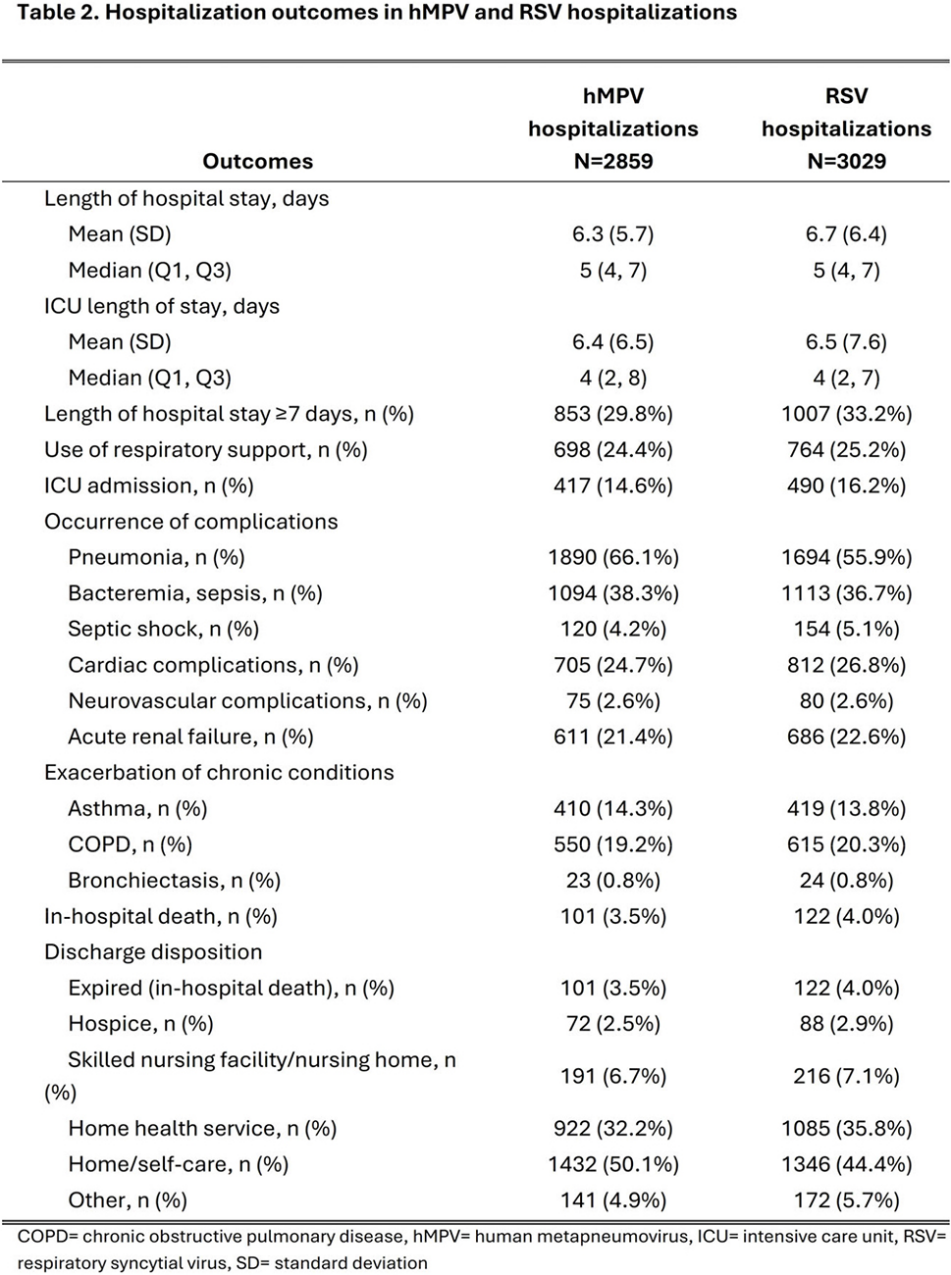

**Methods:**

We performed a retrospective cohort analysis in adults ≥18 years who tested positive for hMPV or RSV by RT-PCR from 7 days prior to 2 days after the date of hospital admission (index date) between 07/01/2011 and 06/30/2024. The outcomes of interest included hospital length of stay, intensive care unit (ICU) admission, occurrence of complications, and in-hospital mortality. Relative risk (RR) of these outcomes in hMPV patients compared to RSV patients with 95% confidence intervals (CI) were estimated using multivariable Poisson regression models with robust error variance.

**Figure:**
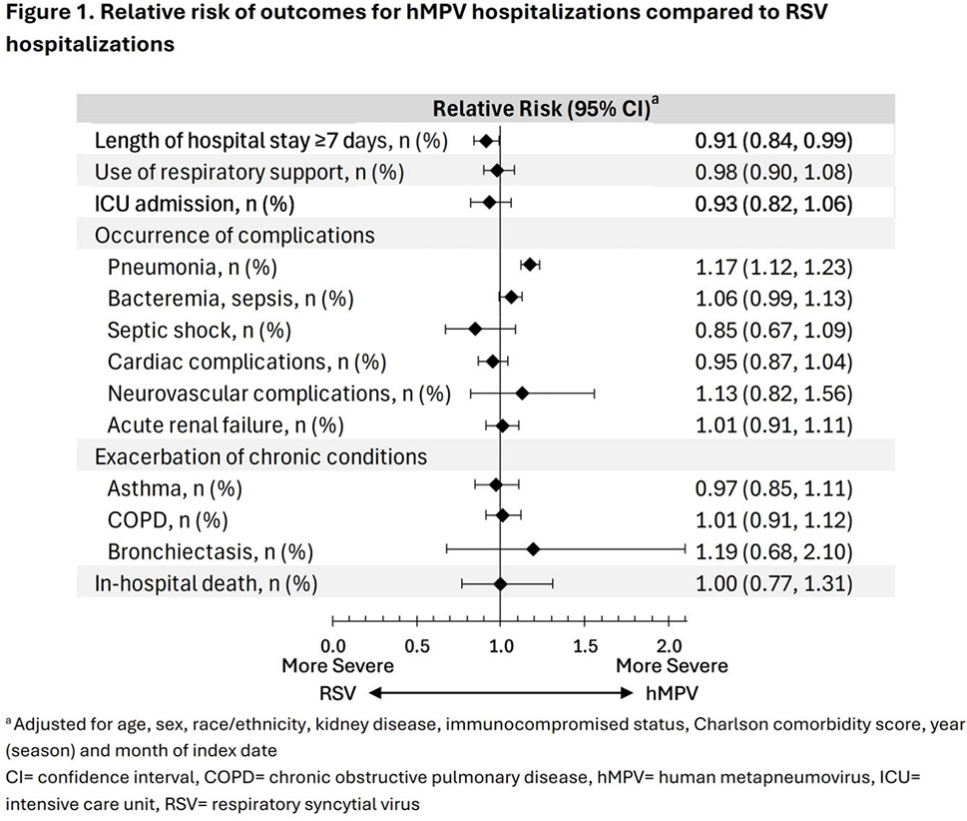

**Results:**

This study included 2859 hMPV-associated and 3029 RSV-associated hospitalizations. The hMPV cohort was similar to the RSV cohort by age (median 74 vs 75 years), sex (59.6% vs 58.0% female) and race/ethnicity (45.9% vs 45.3% non-Hispanic White; Table 1). Approximately 30% of hMPV and RSV hospitalizations had a length of hospital stay ≥7 days and about 15% were admitted to the ICU (Table 2). The risk of an extended hospital stay (≥7 days) was lower in hMPV than in RSV hospitalizations (RR: 0.91 [95% CI: 0.84, 0.99]; Figure 1). Over half of hMPV and RSV hospitalizations had pneumonia diagnoses; this risk was higher in hMPV than in RSV hospitalizations (RR: 1.17 [95% CI: 1.12, 1.23]). Bacteremia/sepsis, cardiac complications, and acute renal failure were observed in 21.4-38.3% of hospitalizations, with similar risk between the two cohorts. No differences were observed in the risk of these outcomes and others, including in-hospital death, between the hMPV and RSV cohorts.

**Conclusion:**

Both hMPV and RSV hospitalizations were associated with substantial and similar healthcare resource utilization and morbidity. The risk of complications and in-hospital mortality in hMPV and RSV hospitalizations was comparable, highlighting the importance of clinical appreciation of severe outcomes associated with hMPV in hospitalized patients.

**Disclosures:**

Emily Rayens, PhD, MPH, AstraZeneca: Grant/Research Support|F2G: Grant/Research Support|GlaxoSmithKline: Grant/Research Support|Moderna: Grant/Research Support Lina S. Sy, MPH, AstraZeneca: Grant/Research Support|Dynavax: Grant/Research Support|GlaxoSmithKline: Grant/Research Support|Moderna: Grant/Research Support Lei Qian, PhD, AstraZeneca: Grant/Research Support|Dynavax: Grant/Research Support|GlaxoSmithKline: Grant/Research Support|Moderna: Grant/Research Support Bradley Ackerson, MD, AstraZeneca: Grant paid to KPSC for work unrelated to this study.|Dynavax: Grant paid to KPSC for work unrelated to this study.|F2G: Grant paid to KPSC for work unrelated to this study.|GSK: Grant paid to KPSC for work unrelated to this study.|Moderna: Grant paid to KPSC for work unrelated to this study.|Pfizer: Grant paid to KPSC for work unrelated to this study. Jaejin An, PhD, AstraZeneca: Grant/Research Support|Bayer: Grant/Research Support|Merck: Grant/Research Support Yi Luo, PhD, AstraZeneca: Grant/Research Support|GlaxoSmithKline: Grant/Research Support|Moderna: Grant/Research Support Xuan Huang, MS, AstraZeneca: Grant/Research Support Jennifer H. Ku, PhD MPH, AstraZeneca: Grant/Research Support|GSK: Grant/Research Support Punam P. Modha, MPH, AstraZeneca: Grant/Research Support|GlaxoSmithKline: Grant/Research Support|Moderna: Grant/Research Support Radha M. Bathala, MS, AstraZeneca: Grant/Research Support|Moderna: Grant/Research Support Sudhir Venkatesan, MPH, PhD, AstraZeneca: Stocks/Bonds (Public Company) Lisa Glasser, MD, AstraZeneca: Stocks/Bonds (Public Company) Daniel Molnar, MSc, AstraZeneca: Stocks/Bonds (Public Company)|GlaxoSmithKline: Stocks/Bonds (Public Company)|Haleon: Stocks/Bonds (Public Company) Richard McNulty, MD, AstraZeneca: Stocks/Bonds (Public Company) Ajoke Sobanjo-ter meulen, MD, MSc, AstraZeneca: Stocks/Bonds (Public Company) Chengbin Wang, MD, PhD, AstraZeneca: Stocks/Bonds (Public Company) Hung Fu Tseng, PhD MPH, AstraZeneca: Grant/Research Support|GlaxoSmithKline: Grant/Research Support|Moderna: Grant/Research Support

